# Identification and in vitro Characterization of a Novel Phage Endolysin that Targets Gram-Negative Bacteria

**DOI:** 10.3390/microorganisms8030447

**Published:** 2020-03-21

**Authors:** Jaewoo Bai, Sangmi Lee, Sangryeol Ryu

**Affiliations:** 1 Department of Food and Animal Biotechnology, Seoul National University, Seoul 08826, Korea; bbbjjjwww@snu.ac.kr; 2 Department of Agricultural Biotechnology, Seoul National University, Seoul 08826, Korea; 3 Division of Applied Food System, Major in Food Science & Technology, Seoul Women’s University, Seoul 01797, Korea; 4 Department of Food and Nutrition, Chungbuk National University, Cheongju, Chungbuk 28644, Korea; sangmilee@chungbuk.ac.kr; 5 Research Institute of Agriculture and Life Sciences, Seoul National University, Seoul 08826, Korea; 6 Center for Food and Bioconvergence, Seoul National University, Seoul 08826, Korea

**Keywords:** cell lysis kinetics, endolysin, flagella-targeting phage, transmembrane domain, secretion

## Abstract

Most double-stranded (ds) DNA phages utilize holin proteins to secrete endolysin for host peptidoglycan lysis. In contrast, several holin-independent endolysins with secretion sequences or signal-arrest-release (SAR) sequences are secreted via the Sec pathway. In this study, we characterized a novel lysis protein (M4Lys) encoded by the dsDNA phage BSPM4, whose lysis function is not dependent on either holin or the Sec pathway in vitro. In silico analysis of M4Lys revealed that it contains a putative virion protein domain and an unusual C-terminal transmembrane domain (TMD). Turbidity reduction assays and liquid chromatography-mass spectrometry using purified peptidoglycan showed that the virion protein domain of M4Lys has peptidoglycan lysis activity. In vitro overproduction of M4Lys in *Escherichia coli* revealed that M4Lys alone caused rapid cell lysis. Treatment of *E. coli* with a Sec inhibitor did not inhibit the lysis activity of M4Lys, indicating that the Sec pathway is not involved in M4Lys-mediated cell lysis. Truncation of the TMD eliminated the cell lysis phenomenon, while production of the TMD alone did not induce the cell lysis. All these findings demonstrate that M4Lys is a novel endolysin that has a unique mosaic structure distinct from other canonical endolysins and the TMD plays a critical role in M4Lys-mediated in vitro cell lysis.

## 1. Introduction

Bacteriophages rely on diverse cell lysis mechanisms to release their progeny from host bacteria. Bacterial lysis by phages can be accomplished in at least two different ways, i.e., peptidoglycan degradation by endolysins or the inhibition of peptidoglycan synthesis [1,2,3]. The majority (96%) of phages described to date are tailed phages that contain a double stranded (ds) DNA genome. Most of these phages achieve host cell lysis by degrading peptidoglycan via the holin-endolysin system while single-stranded RNA or DNA phages inhibit peptidoglycan synthesis [4,5].

Endolysins are peptidoglycan hydrolases that disintegrate integrity of the cell wall by cleaving the peptidoglycan. It is known that endolysins generally cleave glycosidic bonds, peptide bonds, or amide bonds of the peptidoglycan. Holins are small membrane proteins that dissipate membrane potential or make holes in the membrane to allow endolysins to access the peptidoglycan target [4,6]. In the holin-endolysin system, endolysins are produced and accumulate in the cytoplasm in the late stage of the phage lytic cycle. Canonically, holins form holes in the cytoplasmic membrane at a specific time, allowing the endolysin to access peptidoglycan substrates. Peptidoglycan degradation by endolysin results in osmotic imbalance and subsequent rapid cell lysis [7].

In contrast, some endolysins are exported via holin-independent mechanisms. For example, the endolysins of phages P1, 21, and phiKMV, which have N-terminal signal-arrest-release (SAR) domains, are secreted to the periplasm by the Sec pathway. The SAR domain-containing endolysins are anchored to the cytoplasmic membrane in an inactive form until holins dissipate the membrane proton motive force [8,9,10]. The endolysin of *Oenococcus oeni* phage fOg44 (Lys44) contains a typical signal sequence that is converted into the active form after cleavage of the N-terminal signal peptide. The N-terminal region of Lys44 functions as an export signal for the endolysin to translocate to the periplasmic region [11]. The above-mentioned endolysins access the periplasmic region through non-cleavable N-terminal hydrophobic regions or cleavable signal sequences, which function as secretion sequences, and are translocated via Sec machinery. In addition, it has been reported that an auxiliary lysis protein, Gp1, of mycobacteriophage Ms6 is required for efficient host cell lysis by its endolysin, LysA. Gp1 acts as a chaperone-like protein, which is involved in LysA delivery to the peptidoglycan [12].

In this study, we characterized a novel endolysin, M4Lys, identified from the flagella-targeting bacteriophage BSPM4 that was isolated by our group and whose whole genome sequence has been analyzed (accession no. KY620117) [13]. In vitro expression of M4Lys alone in *Escherichia coli* induced rapid cell lysis. The Sec pathway was not responsible for M4Lys-mediated cell lysis in vitro while a C-terminal transmembrane domain (TMD) was indispensable for this cell lysis. Additional cell lysis kinetics studies using various truncations of M4Lys also indicated that the TMD can play an important role for M4Lys-mediated lysis. All these findings suggest that M4Lys can induce cell lysis without the help of holin when it is overproduced in vitro.

## 2. Materials and Methods

### 2.1. In Silico Analysis of the BSPM4 Phage Lysis Cassette

The genome of Salmonella enterica serovar Typhimurium phage BSPM4 was sequenced and annotated previously [13]. The complete genome sequence and annotation results of S. Typhimurium phage BSPM4 are available in GenBank under accession number KY620117 [13]. Domains of M4Lys endolysin were analyzed using BLASTp [14], InterProScan [15], the National Center for Biotechnology Information (NCBI) conserved domain database (CDD) [16], and Phobius [17].

### 2.2. Bacterial Strains, Media, and Growth Conditions

The bacterial strains and plasmids used in this study are listed in Table 1. Bacterial strains were grown in Luria-Bertani medium (Difco, Detroit, MI, USA) at 37 °C with aeration. *Escherichia coli* DH5α and BL21 (DE3) strains were used for cloning and protein expression, respectively. Appropriate antibiotics [50 μg/mL ampicillin (Amp) or 50 μg/mL kanamycin (Kan), final concentration] were used if necessary. When indicated, isopropyl-*β*-thiogalactopyranoside (IPTG) and sodium azide (NaN_3_) were added at 0.1 mM and 1-10 mM, respectively.

### 2.3. Cloning and Expression of the Lysis Proteins

The putative endolysin gene (ORF_38, M4Lys) was amplified by polymerase chain reaction (PCR) with the BSPM4Lys-F-NdeI and BSPM4Lys-R-XhoI primers using phage BSPM4 DNA as template. The PCR product was digested with the respective restriction enzymes and cloned into the pETDuet-1 vector (Novagen, Madison, WI, USA) and sequenced. The recombinant plasmids were, then, used to transform *E. coli* BL21 (DE3) strain and induced to examine cell lysis kinetics. The putative holin gene (ORF_37, M4Holin) was PCR-amplified with the BSPM4Holin-F-NcoI and BSPM4Holin-R-HindIII primers and cloned into the pETDuet-1 as described above. To coexpress M4Lys and M4Holin, both genes were cloned into pETDuet-1 using the primers above. To produce the proteins, 0.1 mM of IPTG was added 3 h after incubation and the lysis kinetics of each strain was observed as previously described [8]. Cell lysis activity of M4Lys was further confirmed with the addition of various concentrations of IPTG (0.0-1.0 mM). Experiments for the lysis kinetics were performed in triplicate. The non-recombinant pETDuet-1 plasmid-harboring *E. coli* BL21 (DE3) strain was used as a control. All the primers used for PCR amplification are listed in Table 2.

### 2.4. Overproduction and Purification of the M4LysΔTMD Protein

To purify a soluble form of M4Lys protein lacking the C-terminal TMD (designated as M4LysΔTMD), the M4LysΔTMD coding gene was cloned into the pET28a vector using the BSPM4Lys-F-NdeI and BSPM4Lys2-R-XhoI primers. In the cloning experiments to generate recombinant plasmids, DNA fragments encoding the putative endolysin gene were inserted between the NdeI and XhoI restriction sites of the pET28a plasmid resulting in an N-terminal His-tag. The recombinant plasmid-harboring *E. coli* BL21 (DE3) strain was incubated for 2 h until an optical density at 600 nm (OD_600_) of 0.5 was reached and was, then, supplemented with 0.5 mM IPTG to produce the M4LysΔTMD protein. After incubation for 3 h, cells were suspended in lysis buffer (20 mM Tris-Cl, 200 mM NaCl, pH 8.0) and sonicated (Branson Ultrasonics, Danbury, CT, USA) to break bacterial cell walls. Sonicated cells were centrifuged at 15,000× *g* for 20 min to obtain the supernatant containing soluble proteins. His-tagged M4LysΔTMD was purified using a Ni-nitrilotriacetic acid Superflow column (Qiagen GmbH, Hilden, Germany) according to the manufacturer’s instructions. The purified protein was visualized with sodium dodecyl sulfate (SDS)-polyacrylamide gel electrophoresis (PAGE) and stored in storage buffer (20 mM Tris-Cl, 200 mM NaCl, 30% glycerol, pH 8.0) after exchanging buffers using a PD Miditrap G-25 column (GE healthcare, Bucks, UK) [19].

### 2.5. Lytic Activity and Host Range Test

Lytic activity of purified M4LysΔTMD was determined using purified *E. coli* peptidoglycan (muropeptides) as substrates. Crude muropeptides of *E. coli* BL21 (DE3) were extracted as described previously with slight modifications [20,21]. In brief, overnight grown *E. coli* cells were harvested and the cells were disrupted by sonication. The cell suspension was then centrifuged at 1400× *g* for 10 min to obtain the crude cell wall lysate. Muropeptides were pelleted by centrifugation at 27,000× *g* for 5 min, resuspended in 4% SDS solution, and boiled for 10 min. Muropeptides were washed using cold distilled water at least 3 times, suspended in Tris-HCl buffer (pH 8.0), and stored at 4 °C until use. Peptidoglycan lysis activity of M4LysΔTMD was examined by measuring a reduction in OD_600_ of the muropeptide solution [21]. To determine the host range, lawns of autoclaved cells of different strains were prepared in 24-well plates. Then, 1 mM of purified M4LysΔTMD was spotted on each lawn and incubated at 37 °C for 12 h. OD_600_ was measured using a SpectraMax i3x (Molecular Devices, Silicon Valley, CA, USA) microplate reader. The reduction of turbidity was calculated by comparing with that of the respective control group without enzyme treatment. The bacterial strains used for host range analysis are listed in Table 3. The efficiency of plating (EOP) was calculated by the comparison of titers between the selected bacteria and the propagation host strain *S.* Typhimurium LT2C [22].

### 2.6. Target Site Identification of M4Lys with HPLC

Muropeptides for HPLC analysis was prepared as described above with slight modifications [23]. Briefly, 400 mL of *E. coli* MG1655 cells were grown exponentially (OD_578_ = 0.6) and harvested by centrifugation at 12,000× *g* for 15 min at 4 °C. The cell pellet was resuspended in 3 mL of ice-cold deionized water (D.W.) and 3 mL of boiling 8% SDS was added dropwise with vigorous stirring within 10 min. The mixture was boiled for 30 min and incubated overnight at room temperature. Muropeptides were collected by ultracentrifugation (130,000× *g*, 60 min at room temperature). The pellet was washed 4 times with 8 mL of D.W. to remove residual SDS. High molecular weight glycogen and covalently bound lipoprotein were cleaved by adding α-amylase (100 μg/mL in 10 mM Tris-HCl, pH 7.5, 2 h at 37 °C) and pronase (200 μg/mL, 90 min at 37 °C), respectively. Samples were mixed with an equal volume (8 mL) of 8% SDS and boiled for 15 min to inactivate the enzymes. Muropeptides were collected by ultracentrifugation and washed 3 times with D.W. to remove SDS from the pellet. The pellet was suspended in 20 mM Tris-HCl buffer (pH 8.0) and stored at 4 °C until use.

Reverse-phase high-performance liquid chromatography (RP-HPLC) analysis was performed as previously described [24] with a few modifications. Total muropeptides were prepared by digesting the purified muropeptides with 20 U mutanolysin (Sigma-Aldrich, St. Louis, MO, USA) at 37 °C in 20 mM Tris-HCl (pH 8.0) for 16 h. The soluble fraction was obtained by centrifugation (20,000× *g*, 15 min at 20 °C) and treated with purified M4LysΔTMD at 25 °C overnight. Samples were heat-inactivated for 10 min and reduced with 5 mg sodium borohydride in 500 mM sodium borate buffer (pH 9.0) for 30 min at room temperature. Borohydride was inactivated by adding 20% phosphoric acid, the pH adjusted to 4, and the sample filtrated using a 0.2 μM filter. Prepared samples were loaded onto a Kinetex C18 column (4.6 × 250 mm, 5u, Phenomenex, Torrance, CA, USA) and a UHPLC C18 column (AJ0-8768, Phenomenex, Torrance, CA, USA) was used as a guard column. RP-HPLC was performed using the Agilent Technologies HPLC 1260 system at the Korea Basic Science Institute (Seoul, South Korea). Samples were injected onto a column preheated to 50 °C and allowed to bind at a flow rate of 0.5 mL/min with a solution of 1% acetonitrile and 0.1% trifluoroacetic acid (TFA) for 10 min. A gradient of 1% to 10% acetonitrile in 0.1% TFA solution was used for elution at a flow rate of 0.5 mL/min for the next 60 min. Absorbance was detected at 205 nm and the fractionated muropeptides were identified by matrix-assisted laser desorption/ionization time-of-flight (MALDI-TOF) mass spectrometry.

### 2.7. Functional Analysis of the M4Lys TMD

To understand the role of M4LysΔTMD and the TMD region, each recombinant gene was PCR-amplified, cloned into the pETDuet-1 dual-expression vector, and expressed with 0.1 mM of IPTG, as described above. The TMD region was truncated from the gene encoding the M4Lys protein with various primers. Primers BSPM4Lys-F-NdeI and BSPM4Lys2-R-XhoI were used to generate the TMD-truncated construct M4LysΔTMD. The following three primer sets were used to generate the partially truncated TMD: BSPM4Lys-F-NdeI and BSPM4Lys5_3-R-XhoI for M4Lys_1–224_, BSPM4Lys-F-NdeI and BSPM4Lys5_4-R-XhoI for M4Lys_1–225_, and BSPM4Lys-F-NdeI and BSPM4Lys6-R-XhoI for M4Lys_1–226_, respectively. All three PCR-amplified constructs were cloned into the pET28a vector and were transformed into *E. coli* BL21 (DE3) cells. Then, recombinant plasmid-harboring *E. coli* cells were used for cell lysis tests with 0.1 mM IPTG induction. Primers used for PCR amplification are listed in Table 2. Experiments for the lysis kinetics were performed in triplicate.

### 2.8. Phylogenetic Analysis and Sequence Alignment of M4Lys

Phylogenetic trees were constructed based on alignment of the M4Lys protein sequence with the amino acid sequences from 37 Gram-negative bacteriophage endolysins available on the NCBI database. Protein sequences were aligned with the Clustal X2 program [25] and the phylogenetic tree was constructed using MEGA7 by the neighbor-joining method with *P* distance values [26]. Amino acid sequence alignments of endolysins from Chi-like phages were conducted with the Clustal X2 algorithm and edited using the GeneDoc tool [25,27].

## 3. Results and Discussion

### 3.1. In Silico Analysis of the BSPM4 Lysis Cassette Showed Two Putative Lysis Genes

**Figure 1:**
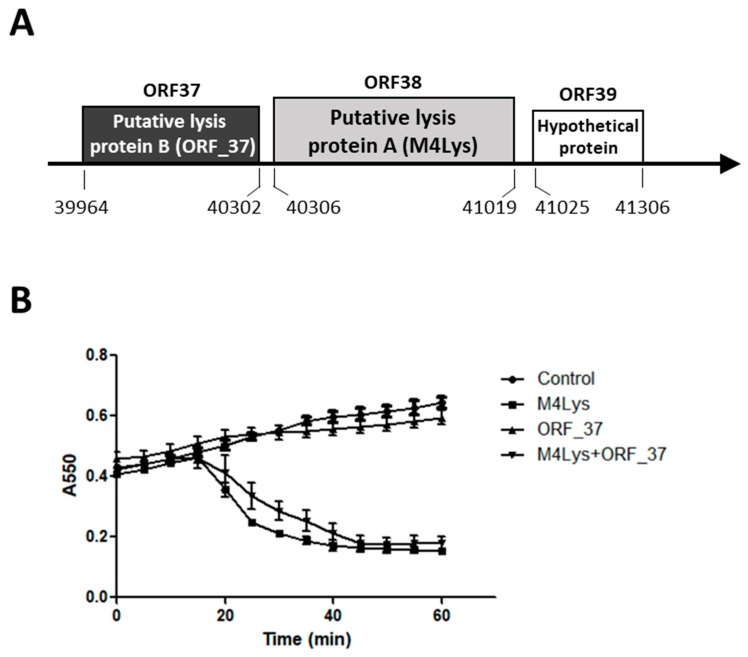
Schematic representation of the phage BSPM4 lysis cassette and analysis of the lysis kinetics by its putative lysis proteins. (**A**) Schematic representation of the phage BSPM4 lysis cassette; (**B**) Growth kinetics of the *E. coli* strains producing the putative lysis protein A (M4Lys) and the putative lysis protein B (ORF_37). Closed circles, empty vector; closed triangles, production of ORF_37; closed squares, production of M4Lys; reverse closed triangles, co-production of ORF_37 and M4Lys. Lysis activity of each gene was investigated by 0.1 mM IPTG induction and the turbidity of the cells was observed. Experiments for the growth kinetics were performed in triplicate. The results of each treatment are represented by the mean ± standard deviation.

The phage BSPM4 genome sequence includes two putative lysis genes in the predicted lysis cassette, putative lysis protein B (ORF_37) and putative lysis protein A (ORF_38). A hypothetical gene (ORF_39) was identified downstream of the two putative lysis genes (Figure 1A). Arrangement of the BSPM4 lysis cassette is similar to those of other flagellotropic *Salmonella* phages such as iEPS5 and FSLSP099 [28,29]. However, the specific function of the putative lysis proteins in host lysis has not yet been characterized.

### 3.2. Only One Putative Lysis Gene Caused Cell Lysis when Overproduced in E. coli

In order to understand which gene encodes the BSPM4 phage endolysin, lysis kinetics were examined by overproducing the putative lysis protein B (ORF_37) and the putative lysis protein A (ORF_38) using an *E*. *coli* expression system. Endolysin is synthesized and accumulated in the cytosol until the holin creates pores in the inner membrane, thus allowing the endolysin to reach its peptidoglycan substrate target [21,30]. Typically, in vitro production of endolysin alone does not trigger cell lysis [21,31]. However, in our study, *E. coli* cells were considerably lysed between 20 and 60 min after putative lysis protein A (M4Lys hereafter) alone was produced by IPTG induction (Figure 1B). Furthermore, M4Lys caused cell lysis more quickly at higher inducer concentrations (data not shown), suggesting that the cell lysis caused by M4Lys is concentration-dependent. Expression of the putative lysis protein B (ORF_37 hereafter) alone showed minimal impact on cell growth compared with the control and did not cause cell lysis (Figure 1B). Coexpression of ORF_37 and M4Lys achieved similar levels of lysis, albeit less rapidly compared with M4Lys expression alone (Figure 1B). Overall, these results suggest that ORF_37 is not essential for host cell lysis in vitro. Slight retardation in lysis rate was observed when the two proteins were produced together compared with M4Lys alone (Figure 1B). We speculate that this could be simply due to the difference in the cellular concentration of M4Lys between the cells producing both M4Lys and ORF_37 proteins simultaneously and those producing M4Lys alone (Figure 1B). However, we cannot exclude the possibility that ORF_37 could control the lysis activity of M4Lys, thus preventing premature lysis.

### 3.3. Sec Machinery is Not Involved in M4Lys-Mediated Lysis In Vitro

Most holin-independent endolysins contain N-terminal signal peptide sequences or SAR sequences essential for their secretion to the periplasm. These sequences often function as secretion sequences for Sec translocase [8,10,11]. However, analysis of the M4Lys protein with various bioinformatics tools (TargetP V1.0, Psort, DGPI, and SignalP 4.1 [32]) did not reveal any distinct N-terminal signal sequence or SAR sequence in the M4Lys protein sequence. A putative signal peptide cleavage site was detected by SignalP 4.1 only when the settings were optimized to increase sensitivity with a cutoff value of 0.140 (data not shown).

**Figure 2:**
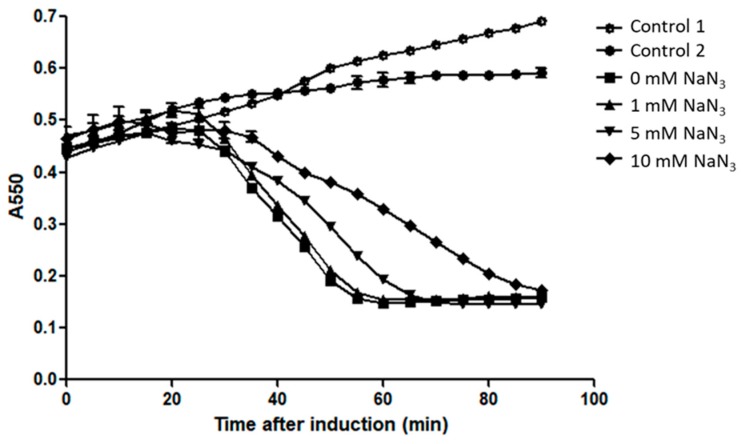
Sec machinery is not involved in M4Lys-mediated cell lysis in vitro. Cell lysis kinetics of M4Lys in the presence of the Sec translocase inhibitor, NaN_3_. In all experiments, expression of M4Lys from pETDuet-1 plasmid in *E. coli* cells was induced by adding 0.1 mM of IPTG, except for the controls. Open circles, no NaN_3_ without IPTG (control 1); closed circles, 10 mM NaN_3_ without IPTG (control 2); closed squares, no NaN_3_; closed triangles, 1 mM NaN_3_; reverse triangles, 5 mM NaN_3_; and diamonds, 10 mM NaN_3._ All experiments for the cell lysis kinetics were performed in triplicate and the results of each treatment are represented by the mean ± standard deviation.

To examine whether Sec machinery is responsible for M4Lys secretion, M4Lys was produced in the presence of NaN_3_, a well-known inhibitor of *E. coli* Sec translocase [10,33]. In this experiment, the host cell lysis kinetics of M4Lys was determined by adding various concentrations of NaN_3_. Increasing concentrations of NaN_3_ delayed cell lysis by M4LysA but did not inhibit cell lysis (Figure 2). The retardation in cell lysis rate could be due to growth inhibition by high concentrations of NaN_3_ (Figure 2). This line of evidence implies that the Sec system might not be involved in M4Lys-mediated cell lysis in vitro.

### 3.4. Domain Analysis of M4Lys

**Figure 3:**
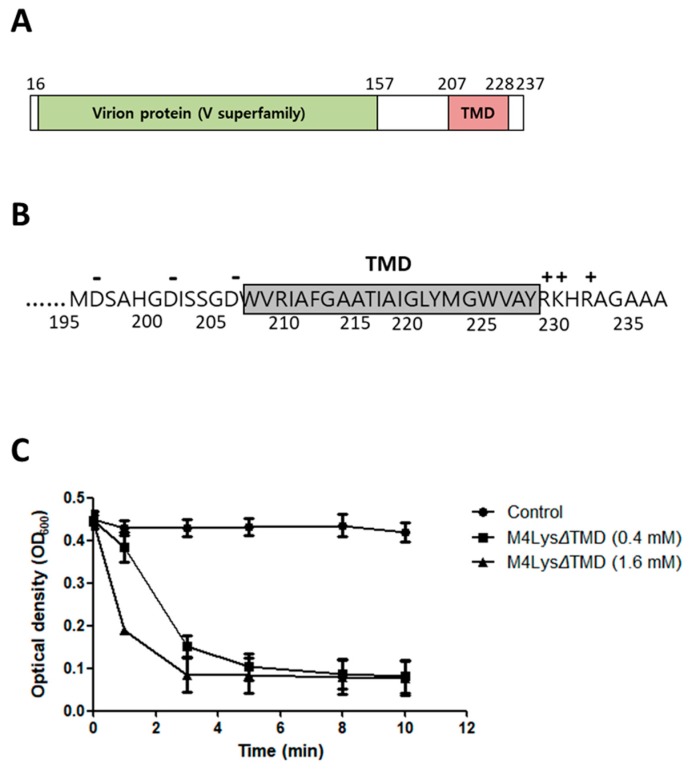
Domain analysis of M4Lys and its peptidoglycan degradation activity. (**A**) Domain analysis of M4Lys. The numbers represent amino acid positions; (**B**) Charge distribution of the TMD region of M4Lys. Positive and negative charges are represented as + and -, respectively, above the amino acid sequences. Numbers under the amino acid sequences indicate amino acid position; (**C**) Turbidity reduction assay of purified M4LysΔTMD using *E. coli* peptidoglycan. Closed circles, buffer treatment (control); closed squares, 0.4 mM M4LysΔTMD treatment; and closed triangles, 1.6 mM M4LysΔTMD treatment. Turbidity reduction assays were performed in triplicate and the results of each treatment are represented by the mean ± standard deviation.

Among the 278 endolysins identified in Gram-negative bacteria phages, the majority of endolysins (93.9%) have a globular structure with a single catalytic domain [34]. The NCBI CDD and sequence search with InterProScan revealed that M4Lys contains a provisional virion protein domain (V superfamily, PHA02564) at the N-terminus (Figure 3A). In addition, Phyre2 analysis revealed that the N-terminus of M4Lys has structural homology (22% of coverage with 82.1% confidence) with the peptidoglycan hydrolase (glycosidase) domain from *S.* Typhimurium flagella protein (FlgJ) (data not shown). Since production of the full-length M4Lys protein caused rapid cell lysis (Figure 1B), we speculated that one of these domains, in particular the virion protein domain, could function as a catalytic domain.

A putative TMD was predicted in the C-terminus of M4Lys using Phobius, Phyre2, and TMHMM algorithms [17,35,36] (Figure 3A). Another TMD was detected with only one bioinformatics program (TMHMM) but not with other programs; hence, this second TMD was not included in our study. It is noteworthy that the TMD structure of M4Lys was similar to membrane-spanning transmembrane helices and it contained the highly charged hydrophilic C-terminus found in holins (Figure 3B) [37]. Given our observation that the expression of M4Lys alone caused rapid cell lysis (Figure 1B) in spite of the absence of an obvious signal sequence, we speculated that the TMD could play an important role in M4Lys-mediated cell lysis in vitro.

### 3.5. Catalytic Activity of M4Lys and Its Target Site Identification

Peptidoglycan lytic activity of the virion protein domain was assessed by deleting the C-terminal transmembrane domain of M4Lys (M4LysΔTMD). The lytic activity of the full-length M4Lys could not be measured since we failed to purify the protein in spite of repeated attempts, presumably due to the rapid cell lysis that occurred upon its induction. M4LysΔTMD of the predicted size (24.5 kDa) was purified (Figure S1) and incubated with the purified peptidoglycan. Optical density rapidly decreased within 4 min, suggesting that the virion protein domain has a peptidoglycan degradation activity (Figure 3C).

**Figure 4:**
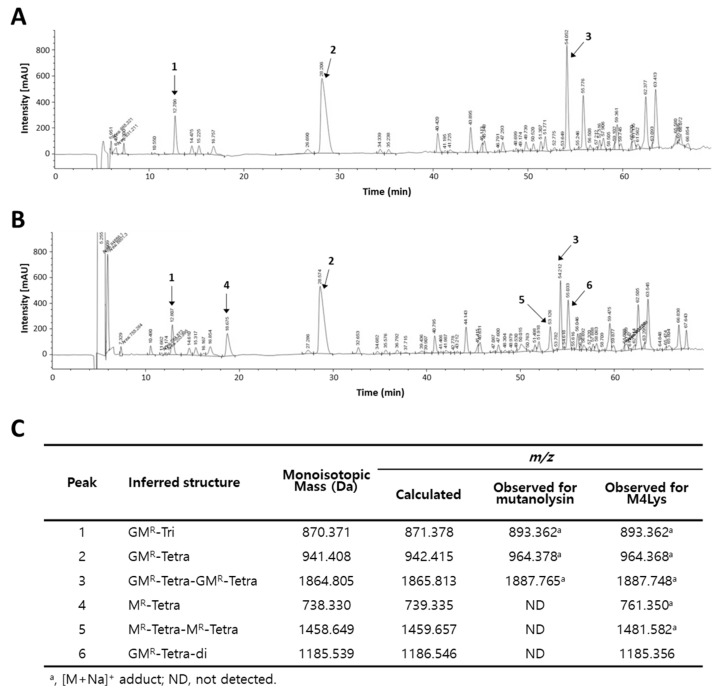
Determination of M4Lys cleavage sites. Analysis of *E. coli* MG1655 peptidoglycan digested by (**A**) mutanolysin and (**B**) mutanolysin with purified M4LysΔTMD. Soluble muropeptides were reduced and analyzed by RP-HPLC coupled to MS. Peaks corresponding to m/z values matching previously identified muropeptides are numbered. The fragmentation pattern (peaks 1, 2, and 3) is typical of Tri (L-Ala-D-Glu-m-DAP), Tetra (L-Ala-D-Glu-m-DAP-D-Ala), and Tetra-Tetra muropeptides, respectively. The fragmentation event leading to the loss of a nonreduced GlcNAc residue (203.078, theoretical mass; peaks 4 and 5) indicates the N-acetylmuramidase activity of M4LysΔTMD. Cleavage of D-Glu-mDAP crosslink (peak 6) shows the endopeptidase activity of M4LysΔTMD; (**C**) Inferred structures, theoretical monoisotopic masses, and theoretical and observed m/z values of individual peaks are tabulated. M^R^, reduced MurNAc; G, GlcNAc; Di, m-DAP (meso-diaminopimelic acid)-D-Ala; Tri, L-Ala-D-Glu-m-DAP; and Tetra, L-Ala-D-Glu-m-DAP-D-Ala.

To date, at least five different groups of peptidoglycan catalytic enzymes have been reported, i.e., *N*-acetylmuramidases (lysozymes), endo-*β*-*N*-acetylglucosaminidases (glycosidases), lytic transglycosylases, endopeptidases, and *N*-acetylmuramoyl L-alanine amidases [38]. To examine the target specificity of the virion protein domain of M4Lys, *E. coli* peptidoglycan (muropeptides) was predigested with a muramidase (mutanolysin) and incubated with purified M4LysΔTMD. Then, the muropeptides were fractionated by RP-HPLC and analyzed by MS (Figure 4). This analysis revealed that a GlcNAc residue (peaks 4 and 5) was absent both from a monomeric disaccharide-tetrapeptide (Tetra; M4, peak 2) and from a dimer of disaccharide-tetrapeptide (Tetra-Tetra; D44, peak 3) (Figure 4), suggesting *N*-acetylglucosaminidase (glycosidase) activity of M4LysΔTMD. This result again supports the Phyre2 prediction that displayed structural homology between the N-terminus of M4Lys and the glycoside hydrolase domain of the flagella protein FlgJ from *Salmonella*. Interestingly, endopeptidase activity of M4Lys could also be inferred from the observation that a crosslinked dimer of disaccharide-tetrapeptide (peak 3) was reduced and a D-Glu-mDAP crosslink cleaved disaccharide (GM^R^-Tetra-Di, peak 6) was generated (Figure 4).

Endolysins often exhibit a broader antimicrobial spectrum than that of the parental phages [38,39,40,41]. Purified M4LysΔTMD showed a lytic spectrum against various Gram-negative bacteria whereas the parental phage BSPM4 could only infect *S*. Typhimurium strains (Table 3).

### 3.6. The M4Lys TMD is Important for Cell Lysis by M4Lys In Vitro

**Figure 5:**
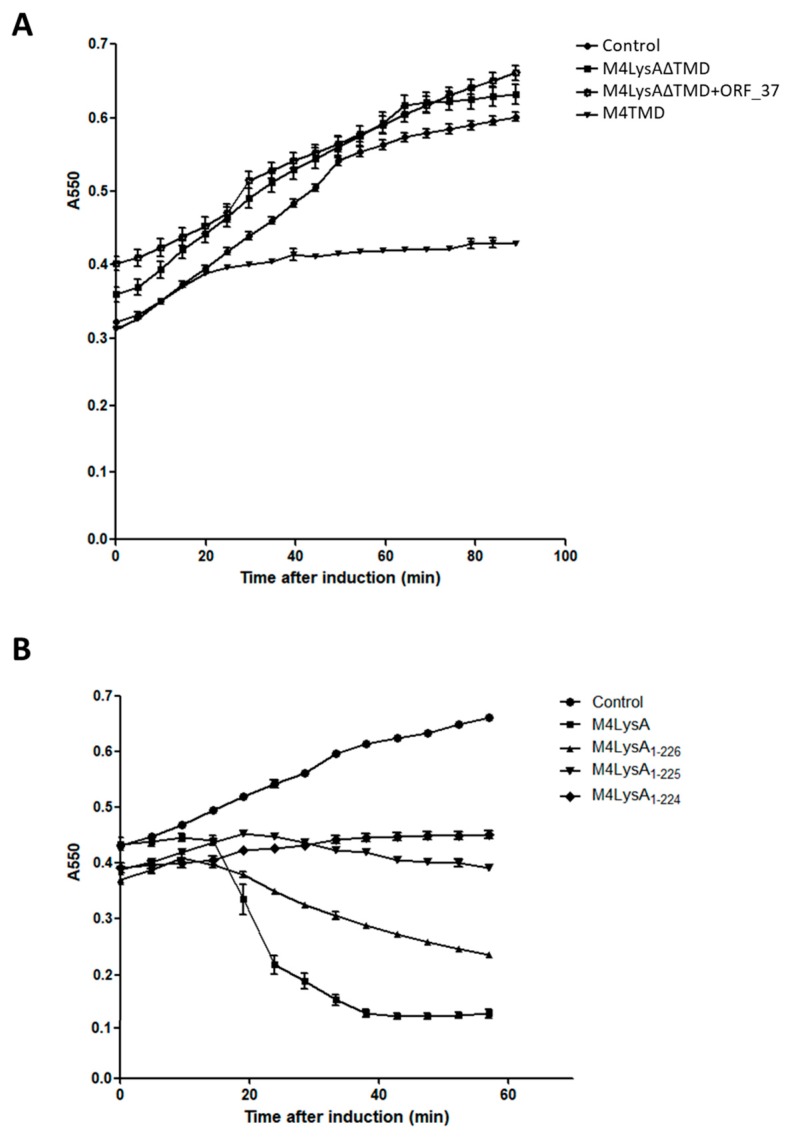
Functional analysis of the M4Lys transmembrane domain. (**A**) Catalytic domain (M4LysΔTMD), C-terminal TMD of M4Lys (M4TMD), and a putative lysis protein B (ORF_37) were produced separately or together in *E. coli* BL21 (DE3) cells. Closed circles, empty vector; closed square, expression of M4LysΔTMD; open square, coexpression of M4LysΔTMD and ORF_37; and reverse closed triangles, expression of TMD; (**B**) Lysis activity of various deletion mutants compared with the full-length M4Lys. Closed circles, empty vector; closed squares, full-length M4Lys; closed triangles, 11 amino acid deletion in TMD of M4Lys (M4Lys_1–226_); reverse closed triangles, 12 amino acid deletion in M4Lys TMD (M4Lys_1–225_); and closed diamonds, 13 amino acid deletion of M4Lys TMD (M4Lys_1–224_). Truncation of 13 amino acids from the M4Lys sequence eliminated the lytic activity. All protein expression was induced by adding 0.1 mM IPTG. Experiments for cell lysis kinetics were performed in triplicate and the results of each treatment are represented by the mean ± standard deviation.

Several lines of evidence previously mentioned suggest that TMD is important for M4Lys-mediated cell lysis in vitro. For instance, in vitro overexpression of M4Lys caused rapid cell lysis (Figure 1B). On the other hand, the expression of M4LysΔTMD alone did not result in cell lysis and the sole expression of TMD exhibited bacteriostatic features (Figure 5A). In addition, expression of M4LysΔTMD with ORF_37 did not trigger cell lysis (Figure 5A). In the topology prediction of TMD using Phobius and Phyre2, the N-terminus region of M4LysA was predicted to face the periplasmic region while the C-terminus region was predicted to be localized in the cytoplasmic region (data not shown). Charge distribution of the TMD region also supported the topology prediction results as the C-terminal region of TMD contains three positively charged amino acids, which are sufficient for orientation of the N-terminal region to the periplasm [42] (Figure 3B).

The importance of the TMD in cell lysis by M4Lys was reinforced by the change in lytic activity of sequential amino acid truncations of M4Lys C-terminus. Lytic activity further decreased with increasing numbers of amino acid residues eliminated. Eventually, cell lysis activity was virtually eliminated when 13 amino acids were deleted from the C-terminus of M4Lys (M4Lys_1–224_) (Figure 5B).

Our current data suggest that the ORF_37 of BSPM4 phage is not required for M4Lys-mediated cell lysis in vitro. However, we cannot currently rule out the possibility that ORF_37 is necessary for optimum cell lysis during BSPM4 phage infection in vivo.

### 3.7. Phylogenetic Analysis of M4Lys

**Figure 6:**
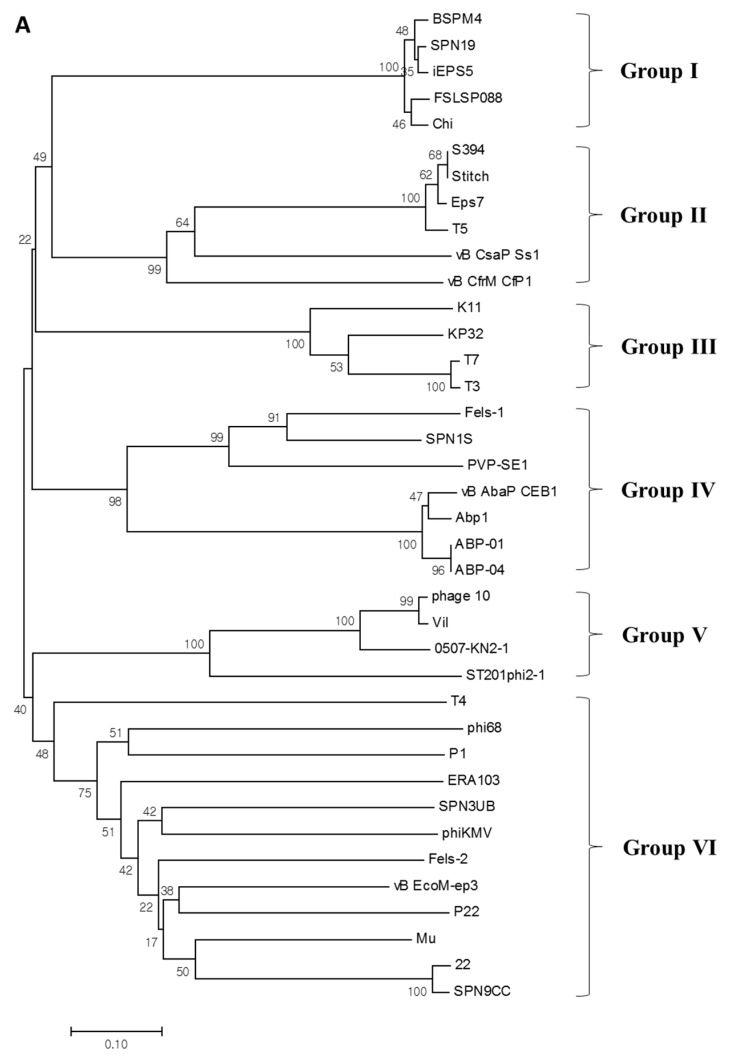

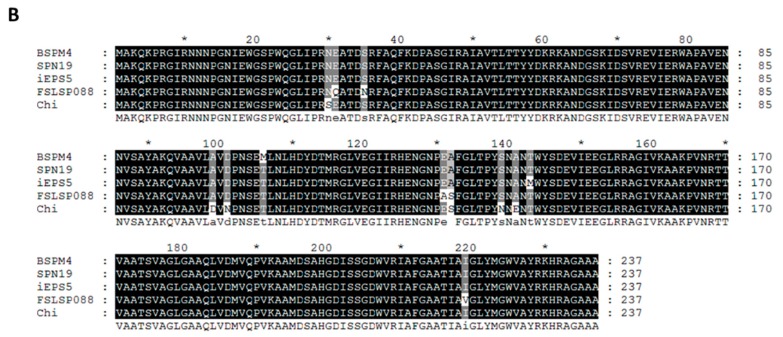
Amino acid sequence alignments of M4Lys with other endolysins. (**A**) Phylogenetic analysis of endolysins from Gram-negative bacteria phages. Each group corresponds to a different functional domain: group I, V superfamily; group II, D-alanyl-D-alanine-carboxypeptidase; group III, *N*-acetyl muramoyl L-alanine amidase; group IV, glycoside hydrolase family 19; group V, *N*-acetylmuramidase; and group VI, glycoside hydrolase family 24, respectively; (**B**) Amino acid sequence alignments of the M4Lys protein of phage BSPM4 with other putative lysis proteins of relevant phages including SPN19, iEPS5, FSLSP088, and Chi. Conserved and identical residues are shaded in gray and black, respectively.

M4LysA has a unique structure distinct from other canonical endolysins. In order to estimate the evolutionary relationship of M4Lys with other endolysins, phylogenetic analysis was conducted with 37 available endolysin sequences identified from phages infecting diverse Gram-negative bacteria. On the basis of the domain composition, M4Lys was classified as a new group of endolysins (group I) along with those originating from flagella-targeting (Chi-like) phages SPN19, iEPS5, FSLSP088, and Chi (Figure 6A) [28,43,44,45]. Interestingly, all five phages that belong to group I infect *Salmonella*. Subsequent BLASTp homology searches and amino acid sequence alignments revealed that the sequence of M4Lys is highly conserved (97% to 99% identity) among Chi-like phages (Figure 6B). These results suggest that flagellotropic Chi-like *Salmonella* phages can share a similar host lysis cassette and that M4Lys is phylogenetically distinct from other endolysins identified from the phages infecting Gram-negative bacteria (Figure 6A).

## 4. Conclusions

In this study, we identified and characterized the cell lysis characteristics of M4Lys, a novel endolysin that causes cell lysis when overproduced in vitro. Of particular note is the unusual structure of M4Lys that contains a putative virion protein domain and a TMD. A series of cell lysis kinetic analyses suggested that M4Lys has peptidoglycan degradation activity and that TMD is indispensable for host cell lysis by M4Lys when M4Lys is overexpressed in vitro. This finding enhances our understanding of the host cell lysis mechanism, especially by the flagella-targeting *Salmonella* phages. However, since this study provided indirect, circumstantial evidence on the potential role of TMD in M4Lys function, further studies are warranted to investigate its specific role in cells. Additionally, we hypothesize that the unique structure of M4Lys could be due to the recombination of two domains and further studies are being conducted in our laboratory to examine the interactions of the two M4Lys domains in an attempt to investigate the mechanism behind the formation of the observed mosaic domain.

## Figures and Tables

**Table 1:** Bacterial strains and plasmids in this study.

Strains	Description	Reference
***Escherichia coli***		
DH5α	F^-^ Φ80*lac*ZΔM15 Δ(*lac*ZYA-*arg*F) U169 *rec*A1 *end*A1 *hsd*R17(r_k_^-^, m_k_^+^) *pho*A *sup*E44 *thi*-1 *gyr*A96 *rel*A1 λ^-^	Invitrogen
BL21 (DE3)	F– *ompT hsdSB* (r_B_– m_B_–) *gal dcm* (DE3)	[18]
**Plasmids**		
pET28a (+)	Expression vector with a hexahistidine tag, Kan^r^	Novagen
pETDuet-1	Dual-expression vector with a hexahistidine tag, Amp^r^	Novagen
pM4LysΔTMD	pET28a-*orf38ΔTMD*_1–206_	This study
M4Lys	pETDuet-1-*orf38*	This study
ORF_37	pETDuet-1-*orf37*	This study
M4Lys-ORF_37	pETDuet-1-*orf37*.*orf38*	This study
M4LysΔTMD	pETDuet-1-*orf38ΔTMD*_1–206_	This study
M4LysΔTMD-ORF_37	pETDuet-1- *orf38ΔTMD*_1–206_.orf37	This study
M4TMD	pETDuet-1-*orf38TMD*_158–237_	This study
M4LysΔTMD-TMD	pETDuet-1- *orf38ΔTMD*_1–206_.*TMD*_158–237_	This study
M4Lys_1–226_	pET28a-*orf38*_1–226_	This study
M4Lys_1–225_	pET28a-*orf38*_1–225_	This study
M4Lys_1–224_	pET28a-*orf38*_1–224_	This study

**Table 2:** Primers used in this study.

Primer	Nucleotide Sequence [5′–3′] ^†^	Restriction Site
BSPM4Lys-F-NdeI	ATG AGG AAA TAA CAT ATG GCT AAA CAG AAG	NdeI
BSPM4Lys-R-XhoI	GAA AAC GAG CGC CTC GAG GAC GCC CGT CTT	XhoI
BSPM4Lys2-R-XhoI	GGC CGC GCC GAA CTC GAG GCG TAC TCA ATC ACC	XhoI
BSPM4Lys5_3-R-XhoI	CTT CCG GTA CTC GAG TCA TCC CAT GTA CAG	XhoI
BSPM4Lys5_4-R-XhoI	CCG ATG CTT CTC GAG CGC TCA CCA TCC CAT	XhoI
BSPM4Lys6-R-XhoI	CCC GGC CCG ATG CTC GAG GTA CTA GAC CCA TCC	XhoI
BSPM4orf37-F-NcoI	CCC GCT AAT TTT TTG TGA GGA CCA TGG GC A TGA GCG AAA TGG AAC G	NcoI
BSPM4orf37-R-HindIII	GTT ATT GCG AAT CCC GCG AAG CTT CTG TTT AGC CAT CGG	HindIII
pETDuet-M4TMD-NcoI-F	CGA GGA AGG TCT GCC CAT GGG GGG CAT CGT TAA G	NcoI
pETDuet-M4TMD-HindIII-R	AAA ACG AGC GCC GCA AGC TTG CCC GTC TTG ATC C	HindIII
Duet-UP1-F	GAT GCG TCC GGC GTA GAG G	-
Duet-DOWN1-R	CGA TTA TGC GGC CGT GTA CAA T	-
Duet-UP2-F	ATT GTA CAC GGC CGC ATA ATC G	-
T7-promoter-F	TAATACGACTCACTATAGGG	-
T7-terminator-R	GCT AGT TAT TGC TCA GCG GTG	-

†, Restriction enzyme sites are underlined.

**Table 3:** Determination of host range of BSPM4 bacteriophage and its endolysin.

Bacterial Strain	BSPM4 (phage)^*^	M4LysΔTMD (endolysin)^#^
***Salmonella enterica***		
**serovar Typhimurium**		
SL1344	CC	++
UK1	CCC	++
LT2	C	+
LT2C	C	+
ATCC14028^†^	C	++
ATCC19586	C	+
ATCC43147	CCC	+
ATCC13076	C	++
DT104	C	+
**serovar Paratyphi**		
A IB 211	-	+
B IB 231	-	++
**serovar Dublin IB 2973**	**-**	**++**
*E. coli*		
BL21	-	+++
***E. coli*** **O157:H7**		
ATCC35150	-	+++
ATCC43890	-	+
ATCC43894	-	+++
ATCC43895	-	++
O157:NM 3204-92	-	++
O157:NM H-0482	-	+
**Gram-negative bacteria**		
*Vibrio fischeri* ES-114 ATCC 700601	-	-
*Pseudomonas aeruginosa* ATCC 27853	-	+++
*Cronobacter sakazakii* ATCC29544	-	+
**Gram-positive bacteria**		
*Staphylococcus aureus* ATCC 29213	-	-
*Staphylococcus epidermis* ATCC 35983	-	-
*Bacillus subtilis* ATCC 23857	-	-
*Bacillus cereus* ATCC 14579	-	-

†, ATCC, American Type Culture Collection. *, CCC, efficiency of plating (EOP) of 2 to 1.5; CC, EOP of 1.5 to 1; C, EOP of 1 to 0.5; −, not susceptible to phages. #, Degree of OD reduction; +++, 0.4 to 0.6; ++, 0.3 to 0.4; +, 0.2 to 0.3; -, no reduction.

## Supplementary Materials

The following are available online at https://www.mdpi.com/2076-2607/8/3/447/s1, Figure S1: Expression and purification of M4LysΔTMD.
