# Supplementary material for: Identification and in vitro Characterization of a Novel Phage Endolysin that Targets Gram-Negative Bacteria
Source: Microorganisms. 2020 Mar 21;8(3):447. doi: 10.3390/microorganisms8030447 (PMC7143992; doi:10.3390/microorganisms8030447)
Supplement: Supplementary file 1 [file microorganisms-08-00447-s001.pdf]

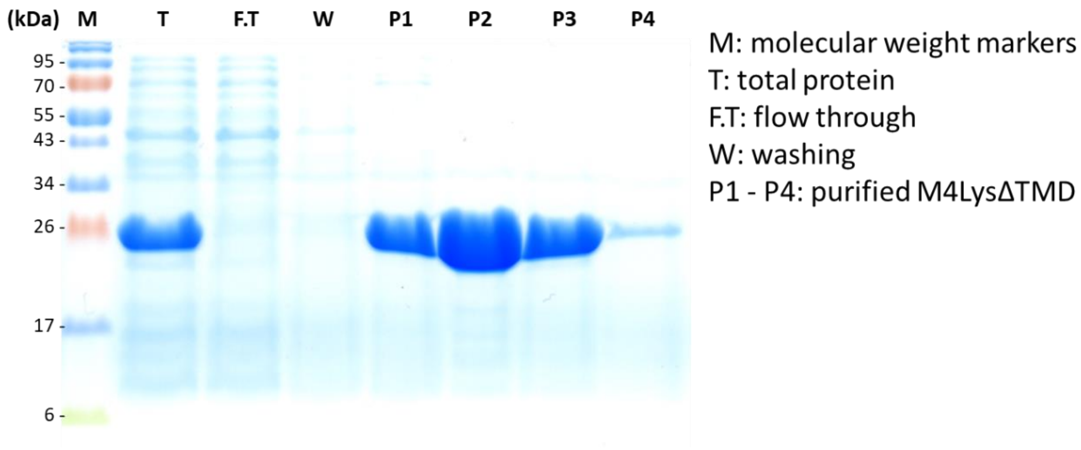

Figure S1. Expression and purification of M4LysΔTMD. (A) Total proteins from *Escherichia coli* BL21(DE3) cells containing the recombinant plasmid after inducing by 0.5 mM of isopropyl-*β*-thiogalactopyranoside (IPTG) (T). The soluble form of recombinant M4LysΔTMD protein (24.5 kDa) was purified using affinity chromatography (P1-P4).
